# Supplementary material for: Speciation and Antibiotic Susceptibilities of Coagulase Negative Staphylococci Isolated from Ocular Infections
Source: Antibiotics (Basel). 2021 Jun 16;10(6):721. doi: 10.3390/antibiotics10060721 (PMC8234609; doi:10.3390/antibiotics10060721)
Supplement: Supplementary file 1 [file antibiotics-10-00721-s001.zip › Kowalski File S1 DNA sequence Identification of CoNS 04132021.pdf]

# File S1. (Kowalski) DNA sequence Identification of CoNS

DNA Sequencing Full Protocol:

Part 1: DNA Extraction

1. Isolate is grown overnight on 5% TSA Blood Agar or Mannitol Agar.
2. 25 µL of DNA Quick Extract Solution (Lucigen Corp., Middleton, WI) is added to a 0.2 mL tube and kept on ice. 25 µL for each isolate. The 0.2 mL tubes come in strips of 8 that are connected.
3. Approximately 1 colony of bacteria is lifted from the agar and mixed into the 25 µL of Quick Extract Solution.
4. The tubes are then placed in an Applied Biosystems 2720 Thermal Cycler (Applied Biosystems, Foster City, CA) according to standard DNA Extraction protocol, which includes 6 minutes at 65°C and 2 minutes at 98°C.

Part 2: PCR Amplification of DNA

1. DNA extracts are removed and placed on ice.
2. A 1.5 mL microcentrifuge tube is used where a PCR reagent cocktail will be mixed.
3. The cocktail contains 41.5 µL DNAse free and RNAse free $ddH_2O$, 5 µL of 10x ThermoPol Buffer solution and other reagents listed below from New England Biolabs (New England Biolabs, Ipswich, MA), 0.5 µL of each degenerate primer as according to the protocol of Poyart et. al (Poyart, C., Quesne, G., Boumaila, C., Trieu-Cuot, P. (2001) "Rapid and accurate species-level identification of coagulase-negative staphylococci by using the sodA gene as a target." J. Clin. Microbiol. 39:4296-4301). Primers were ordered from Integrated DNA Technologies (Coralville, IA). It also contains 0.5 µL $MgCl_2$, 0.5 µL DNTPs, and 0.5 µL Taq Polymerase per isolate for a total of 49 µL per isolate (New England Biolabs, Ipswich, MA). This cocktail can be multiplied to match the number of isolates being run in PCR. All reagents excluding the water are stored at -20°C.
4. Once the cocktail is completed and mixed, 49 µL of cocktail will be pipeted into a new group of 0.2 mL tubes labeled for PCR.
5. 1 µL of the DNA quick extracts will be added to the 49 µL of cocktail to make a PCR solution of 50 µL.
6. The PCR tubes are placed in the thermal cycler where they will undergo 5 minutes at 94°C, then 32 cycles of 30s at 94°C, 30s at 37°C, and 30s at 72°C followed by 7 minutes at 72°C.

7. Once finished, the PCR tubes will be kept on ice or stored at 4°C.

Part 3: Confirmation of DNA Amplification

1. To confirm amplification of DNA, the PCR products must be run on an agarose gel and stained for DNA.
2. 0.8g of agarose is added to 100 mL of 1x Sodium Borate buffer and microwaved for approximately 1.5 minutes to dissolve the agarose.
3. After dissolving, 1.5 μL of SYBR SAFE (Invitrogen – Thermo Fisher Scientific, Waltham, MA) is added to the solution and mixed.
4. The solution is formed into a gel.
5. To test for DNA, a 1 kB ladder is used against a combination of 2 μL of 6X Dye and 5 μL of PCR product.
6. The gel is run on 120 V current for Approximately 30 minutes in 1x Sodium Borate buffer.
7. Once finished, the gel is removed and analyzed for DNA bands under UV light using the Carestream system (Carestream, New Haven, CT).
8. A picture is captured, and isolates that match the Ladder's DNA bands can be set aside for PCR purification. Isolates that did not produce DNA bands must be redone.

Part 4: PCR Purification

1. If PCR products are confirmed on agarose gel, they can be purified to be sent for sequencing.
2. The Qiagen (Hilden, Germany) MinElute PCR Purification Kit is used to purify the PCR products.
3. 4 volumes of PB buffer is added to the 1 remaining volume of PCR product, in this case, 225 μL of PB buffer is added to the 45 μL of remaining PCR product in a minelute column.
4. The minelute columns are centrifuged for 1 minute at 14,000 RPM (same for all centrifugations in this process) and the remaining flowthrough is discarded.
5. 750 μL of PE buffer is then added to the minelute columns and centrifuged again. The flowthrough is discarded.
6. After discarding flowthrough, minelute columns are immediately centrifuged again to remove any remaining PE buffer.
7. After this centrifugation, the bottoms of the minelute columns are discarded and the filter portions are placed inside 1.5 mL microcentrifuge tubes.

8. In these tubes, 20 µL of EB buffer is added to the columns and allowed to stand for one minute. After the minute is up, the tubes will be centrifuged once more and the top filter of the minelute column is discarded, leaving the desired DNA product in the 2mL microcentrifuge tube, which is then closed and placed on ice.

Part 5: Sequencing

1. Sequencing was performed by the University of Pittsburgh Genomic Core Facility.
2. Sequencing was returned via a FinchTV (Perkin Elmer, Waltham, MA) software file.
3. We used the NCBI BLASTN (Altschul, S.F., Gish, W., Miller, W., Myers, E.W. & Lipman, D.J. (1990) "Basic local alignment search tool." J. Mol. Biol. 215:403-410.) software to input and analyze the sequence from the FinchTV software file.
4. The NCBI BLASTN Software outputs an ID match based on the sequence.
