# Supplementary material for: Speciation and Antibiotic Susceptibilities of Coagulase Negative Staphylococci Isolated from Ocular Infections
Source: Antibiotics (Basel). 2021 Jun 16;10(6):721. doi: 10.3390/antibiotics10060721 (PMC8234609; doi:10.3390/antibiotics10060721)
Supplement: Supplementary file 1 [file antibiotics-10-00721-s001.zip › Kowalski Table S1 CoNS ID 3 methods 04132021.pdf]

Table S1. (Kowalski) Supplemental Data of 150 CoNS Isolates and Speciationby API Staph, Biolog, and DNA Sequencing

[E – Endophthalmitis; K – Keratitis; B – Blepharitis/Conjunctivitis]

	STAPH ID KIT		BIOLOG ID		DNA Sequencing
Lab #	Species		Species		Species
E753	S. epidermidis		S. epidermidis		S. epidermidis
E757	S. epidermidis		S. epidermidis		S. epidermidis
E759	S. epidermidis		S. epidermidis		S. epidermidis
E761	S. lugdunensis		S. epidermidis		S. epidermidis
E762	S. epidermidis		S. epidermidis		S. epidermidis
E767	S. epidermidis		S. epidermidis		S. epidermidis
E769	S. epidermidis		S. epidermidis		S. epidermidis
E771	S. epidermidis		S. epidermidis		S. epidermidis
E782	S. epidermidis		S. epidermidis		S. epidermidis
E784	S. epidermidis		S. epidermidis		S. epidermidis
E785	S. aureus		S. epidermidis		S. epidermidis
E786	S. epidermidis		S. epidermidis		S. epidermidis
E787	S. epidermidis		S. epidermidis		S. epidermidis
E790	S. epidermidis		S. epidermidis		S. epidermidis
E791	S. lugdunensis		S. lugdunensis		S. epidermidis
E792	S. epidermidis		S. epidermidis		S. epidermidis
E794	S. epidermidis		S. epidermidis		S. epidermidis
E796	S. epidermidis		S. epidermidis		S. epidermidis
E797	S. epidermidis		S. epidermidis		S. epidermidis
E798	S. epidermidis		S. epidermidis		S. epidermidis
E801	S. epidermidis		S. epidermidis		S. epidermidis
E803	S. epidermidis		S. epidermidis		S. epidermidis
E804	S. hominis		S. hominis		S. hominis
E807	S. hominis		S. lugdunensis		S. lugdunensis
E810	S. epidermidis		S. epidermidis		S. epidermidis
E811	S. epidermidis		S. epidermidis		S. epidermidis
E814	S. epidermidis		S. lugdunensis		S. lugdunensis
E816	S. epidermidis		S. epidermidis		S. epidermidis
E817	S. hominis		S. lugdunensis		S. lugdunensis
E820	S. epidermidis		S. epidermidis		S. epidermidis
E822	S. epidermidis		S. epidermidis		S. epidermidis
E823	S. epidermidis		S. epidermidis		S. epidermidis
E825	S. epidermidis		S. epidermidis		S. epidermidis
E826	S. epidermidis		S. epidermidis		S. epidermidis
E827	S. epidermidis		S. epidermidis		S. epidermidis
E831	S. epidermidis		S. epidermidis		S. epidermidis
E836	S. epidermidis		S. epidermidis		S. epidermidis
E839	S. epidermidis		S. epidermidis		S. epidermidis
E840	S. epidermidis		S. epidermidis		S. epidermidis
E844	S. epidermidis		S. epidermidis		S. epidermidis

E847	S. epidermidis		S. epidermidis		S. epidermidis
E848	S. epidermidis		S. epidermidis		S. epidermidis

E852	S. epidermidis		S. epidermidis		S. epidermidis
E853	S. epidermidis		S. epidermidis		S. epidermidis
E855	S. epidermidis		S. epidermidis		S. epidermidis
E863	S. haemolyticus		S. haemolyticus		S. haemolyticus
E872	S. capitis		S. epidermidis		S. epidermidis
E874	S. epidermidis		S. epidermidis		S. epidermidis
E877	S. epidermidis		S. epidermidis		S. epidermidis
E878	S. epidermidis		S. epidermidis		S. epidermidis
K2549	S. epidermidis		S. epidermidis		S. epidermidis
K2551	S. epidermidis		S. epidermidis		S. epidermidis
K2590	S. epidermidis		S. epidermidis		S. epidermidis
K2624	S. epidermidis		S. epidermidis		S. epidermidis
K2663	S. epidermidis		S. epidermidis		S. epidermidis
K2671	S. epidermidis		S. epidermidis		S. epidermidis
K2699	S. epidermidis		S. epidermidis		S. epidermidis
K2705	S. epidermidis		S. epidermidis		S. epidermidis
K2708	S. epidermidis		S. epidermidis		S. epidermidis
K2714	S. epidermidis		S. epidermidis		S. epidermidis
K2715	S. epidermidis		S. epidermidis		S. epidermidis
K2724	S. epidermidis		S. epidermidis		S. epidermidis
K2725	S. epidermidis		S. epidermidis		S. epidermidis
K2737	S. caprae		S. pasteuri		S. caprae
K2743	S. epidermidis		S. epidermidis		S. epidermidis
K2748	S. epidermidis		S. epidermidis		S. epidermidis
K2755	S. epidermidis		S. epidermidis		S. epidermidis
K2761	S. aureus		S. epidermidis		S. epidermidis
K2770	S. hominis		S. lugdunensis		S. lugdunensis
K2776	S. epidermidis		S. epidermidis		S. epidermidis
K2788	S. epidermidis		S. epidermidis		S. epidermidis
K2795	S. epidermidis		S. epidermidis		S. epidermidis
K2798	S. epidermidis		S. epidermidis		S. epidermidis
K2801	S. hominis		S. hominis ss novobioseptI-cus		S. epidermidis
K2820	S. epidermidis		S. epidermidis		S. epidermidis
K2830	Micrococcus spp		S. capitis ss urealyticus		S. pettenkoferi
K2838	S. caprae		S. capitis ss urealyticus		S. capitis
K2861	S. epidermidis		S. epidermidis		S. epidermidis
K2888	S. caprae		S. capitis ss urealyticus		S. aureus
K2894	S. epidermidis		S. epidermidis		S. epidermidis
K2907	S. epidermidis		S. epidermidis		S. epidermidis
K2911	S. epidermidis		S. epidermidis		S. epidermidis
K2912	S. warneri		S. pasteuri		S. warneri
K2913	S. epidermidis		S. epidermidis		S. epidermidis
K2919	S. epidermidis		S. epidermidis		S. epidermidis

K2929	S. epidermidis		S. epidermidis		S. epidermidis
K2930	S. epidermidis		S. epidermidis		S. epidermidis
K2941	S. epidermidis		S. epidermidis		S. epidermidis
K2964	S. epidermidis		S. epidermidis		S. epidermidis
K2978	S. lugdunensis		S. capitis ss urealyticus		S. capitis
K2994	S. epidermidis		S. epidermidis		S. epidermidis
K2997	S. epidermidis		S. epidermidis		S. epidermidis
K2998	S. epidermidis		S. epidermidis		S. epidermidis
K3001	S. warneri		S. epidermidis		S. epidermidis
K3018	S. epidermidis		S. epidermidis		S. epidermidis
K3034	S. epidermidis		S. epidermidis		S. epidermidis
K3044	S. epidermidis		S. epidermidis		S. epidermidis
K3076	S. epidermidis		S. hominis ss novobiosepti-cus		S. epidermidis
K3098	S. epidermidis		S. hominis ss novobiosepti-cus		S. epidermidis
K3103	S. epidermidis		S. epidermidis		S. epidermidis
B843	S. epidermidis		S. epidermidis		S. epidermidis
B852	S. warneri		S. pasteuri		S. warneri
B861	S. epidermidis		S. epidermidis		S. epidermidis
B864	S. epidermidis		S. epidermidis		S. epidermidis
B875	S. epidermidis		S. epidermidis		S. epidermidis
B907	S. epidermidis		S. epidermidis		S. epidermidis
B914	S. epidermidis		S. epidermidis		S. epidermidis
B921	S. epidermidis		S. epidermidis		S. epidermidis
B947	S. haemolyticus		S. haemolyticus		S. haemolyticus
B948	S. epidermidis		S. epidermidis		S. epidermidis
B949	S. epidermidis		S. epidermidis		S. epidermidis
B1006	S. aureus		S. capitis ss urealyticus		S. warneri
B1070	S. aureus		S. epidermidis		S. epidermidis
B1083	S. aureus		S. epidermidis		S. epidermidis
B1107	S. aureus		S. lugdunensis		S. haemolyticus
B1110	S. epidermidis		S. epidermidis		S. haemolyticus
B1119	S. lugdunensis		S. lugdunensis		S. epidermidis
B1177	S. epidermidis		S. epidermidis		S. epidermidis
B1267	S. epidermidis		S. epidermidis		S. epidermidis
B1268	S. epidermidis		S. epidermidis		S. epidermidis
B1309	S. epidermidis		S. epidermidis		S. epidermidis
B1344	S. lugdunensis		S. lugdunensis		S. lugdunensis
B1363	S. epidermidis		S. epidermidis		S. epidermidis
B1369	S. epidermidis		S. epidermidis		S. epidermidis
B1379	S. capitis		S. capitis ss urealyticus		S. capitis
B1388	S. epidermidis		S. hominis hominis		S. hominis
B1389	S. aureus		S. saprophyticus		S. pasteuri
B1390	S. aureus		S. caprae		S. caprae
B1439	S. epidermidis		S. epidermidis		S. epidermidis
B1461	S. hominis		S. warneri		S. haemolyticus

B1472	S. epidermidis		S. epidermidis		S. epidermidis
B1483	S. epidermidis		S. epidermidis		S. epidermidis
B1522	S. epidermidis		S. epidermidis		S. epidermidis
B1523	S. caprae		S. capitis ss urealyticus		S. caprae
B1541	S. warneri		S. haemolyticus		S. epidermidis
B1542	S. chromogenes		S. epidermidis		S. epidermidis
B1543	S. epidermidis		S. epidermidis		S. epidermidis
B1547	S. haemolyticus		S. haemolyticus		S. haemolyticus
B1554	S. epidermidis		S. epidermidis		S. epidermidis
B1560	S. epidermidis		S. epidermidis		S. epidermidis
B1568	S. epidermidis		S. epidermidis		S. epidermidis
B1637	S. epidermidis		S. epidermidis		S. epidermidis
B1651	S. hominis		S. epidermidis		S. epidermidis
B1656	S. epidermidis		S. epidermidis		S. epidermidis
B1677	S. cohnii ssp urealyticus		S. cohnii ss urealyticus		S. cohnii
B1695	S. epidermidis		S. epidermidis		S. epidermidis
B1739	S. epidermidis		S. epidermidis		S. epidermidis
B1767	S. epidermidis		S. hominis		S. hominis
B1725	S. sciuri		S. lugdunensis		S. lugdunensis
B1812	S. epidermidis		S. epidermidis		S. epidermidis
